# Prevention of sexual transmission of mpox: a systematic review and qualitative evidence synthesis of approaches

**DOI:** 10.1080/23744235.2024.2364801

**Published:** 2024-07-03

**Authors:** Sara Paparini, Isabelle Whelan, Chikondi Mwendera, Rosalie Hayes, Ismael Maatouk, Rosamund Lewis, Mateo Prochazka Nunez, Antons Mozalevskis, Teodora Wi, Chloe Orkin

**Affiliations:** a SHARE Collaborative, Wolfson Institute of Population Health, Queen Mary University of London, London, UK; b Department of Infection and Immunity, Barts Health NHS Trust, London, UK; c Institute of Infection, Veterinary and Ecological Sciences, University of Liverpool, Liverpool, UK; d Department of Global HIV, Hepatitis and Sexually Transmitted Infections Programmes, World Health Organization, Geneva, Switzerland; e Health Emergencies Programme, World Health Organization, Geneva, Switzerland; f SHARE Collaborative, Blizard Institute, Queen Mary University of London, London, UK

**Keywords:** Mpox, MPXV, prevention, men who have sex with men, sexual transmission

## Abstract

**Background:**

The ongoing multi-country mpox outbreak in previously unaffected countries is primarily affecting sexual networks of men who have sex with men. Evidence is needed on the effectiveness of recommended preventive interventions. To inform WHO guidelines, a systematic review and qualitative evidence synthesis were conducted on mpox preventive behavioural interventions to reduce: (i) sexual acquisition; (ii) onward sexual transmission from confirmed/probable cases; and (iii) utility of asymptomatic testing.

**Methods:**

Medline, EMBASE, PubMed, Cochrane and WHO trial databases, grey literature and conferences were searched for English-language primary research published since 1 January 2022. A reviewer team performed screening, data extraction and bias assessment. A qualitative thematic synthesis explored views and experiences of engagement in prevention in individuals at increased risk.

**Results:**

There were 16 studies: 1 on contact-tracing, 2 on sexual behaviour, and 13 on asymptomatic testing. Although MPXV was detected in varying proportions of samples (0.17%–6.5%), the testing studies provide insufficient evidence to fully evaluate this strategy. For the qualitative evidence synthesis, four studies evaluated the experiences of most affected communities. Preferences about preventive interventions were shaped by: mpox information; the diversity of sexual practices; accessibility and quality of mpox testing and care; and perceived cost to wellbeing.

**Conclusions:**

Evidence on the effectiveness of interventions to prevent the sexual transmission of mpox remains scarce. Limited qualitative evidence on values and preferences provides insight into factors influencing intervention acceptability. Given global and local inequities in access to vaccines and treatment, further research is needed to establish the effectiveness of additional interventions.

## Introduction

In May 2022, near-simultaneous rapidly evolving outbreaks of mpox (formerly monkeypox) due to clade IIb monkeypox virus (MPXV) were reported from several countries not historically affected by sustained mpox transmissions. The World Health Organization (WHO) declared a public health emergency of international concern in July 2022 [1]. As of 31 January 2024, a total of 93,921 laboratory confirmed cases and 662 probable cases, including 179 deaths, have been reported to WHO from 117 countries in all six WHO regions [2]. In contrast to previous outbreaks, this multi-country mpox outbreak primarily emerged in sexual networks of sexually active men who have sex with men (MSM), and approximately 50% of persons affected are also living with HIV [3,4]. Large case series and studies (predominantly in MSM) described differences in clinical features and high propensity for transmission through mucosal (especially rectal) contact during this recent outbreak [5,6]. In addition, sexual transmission of the more virulent clade I MPXV in central Africa has also been documented for the first time [7].

In accordance with the International Health Regulations (IHR) [8], WHO and other public health agencies issued a series of temporary recommendations [9]. People with or at risk of mpox were advised to: isolate; avoid sexual and skin-to-skin contact while symptomatic; reduce number of sex partners, particularly in certain contexts (e.g. sex-on-premises venues); and use personal protective measures [10].

As well as the above recommendations, preventive interventions could include changes in types of sexual practices; evaluation with partners (e.g. asking about symptoms or lesions); seeking testing and care as needed, and, for confirmed cases, covering any lesions, disclosure to partners, and contact tracing. Asymptomatic screening in people with other sexually transmitted infections is another potential measure to assess the status of the outbreak and prevent onward transmission.

To address evidence gaps about preventive interventions, the WHO Health Emergencies Programme commissioned a systematic review addressing three population-intervention-comparison-outcomes (PICO) questions about sexual transmission of mpox:

1. Do preventive interventions reduce the risk of mpox infection?
2. Do prevention interventions reduce the risk of onward transmission from a person with mpox?
3. Does testing asymptomatic individuals at increased risk of mpox (without known exposure)reduce onward transmission?

The review concentrates specifically on prevention strategies related to sexual transmission but does not include vaccines and vaccination campaigns. This is due to the limited evidence available on the effectiveness of non-vaccine preventive (risk-limiting) interventions in sexual transmission or the natural history of mpox.

Additionally, we conducted a separate qualitative evidence synthesis to contextualise the findings from the systematic review and understand the views on and experiences of preventive interventions among members of most affected communities. Methods and findings from the two reviews are presented individually, while complementing each other in the Discussion.

## Materials and methods

### Systematic review

This systematic review is based on the protocol registered PROSPERO (ref: CRD420234259060) and reported in accordance with the PRISMA 2020 checklist.

#### Eligibility criteria

In consultation with information specialists, we conducted a single search covering all questions (Appendix A). Primary research studies, case series and letters containing reports on primary research published since January 2022 were included. Individual case reports, modelling and animal studies, and pre-prints were excluded. Papers related to vaccine interventions were excluded because a separate review is in process to support guidelines on vaccination. The search was limited to English language due to time constraints. Inclusion and exclusion criteria are described in Appendix A.

#### Information sources

The following databases were searched for papers from 1 January 2022 to 4 May 2023: Medline, EMBASE and PubMed (last searched 3 May 2023); Cochrane Library (for systematic reviews) and WHO trials (last searched 4 May 2023); PROSPERO reviews database. We reviewed available conference abstracts from 2022 and 2023for relevant meetings: International AIDS Conference; European AIDS Conference; Conference on Retroviruses and Opportunistic Infections; ID Week.^1^ Backward and forward citation tracking was conducted for all included papers, last conducted on 6 July 2023 using Web of Science. The search was updated on 11 January 2024.

#### Search strategy

For the full search strategies for all databases, see Appendix B.

#### Selection process

**Figure 1.**
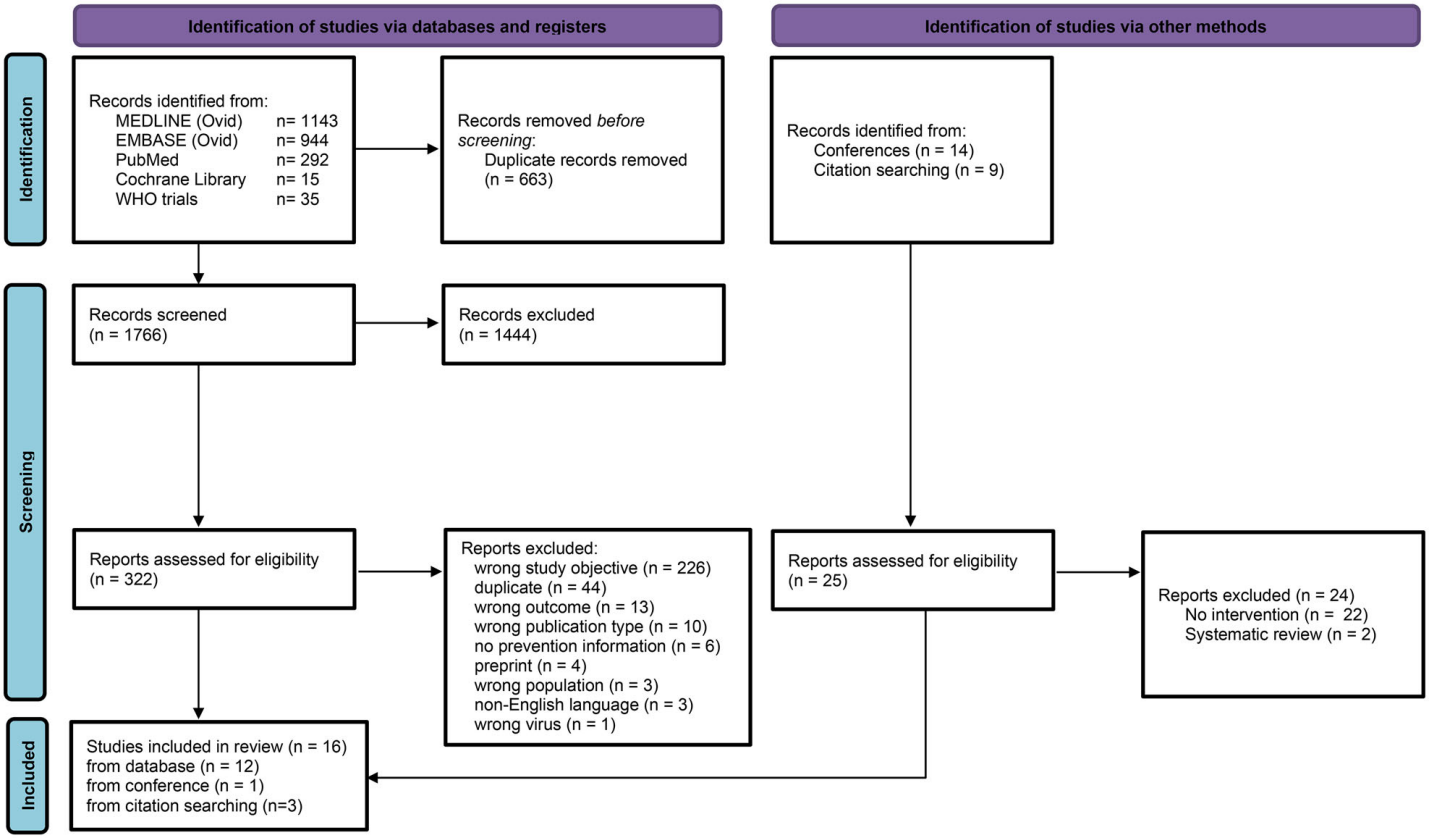
Systematic review of interventions to prevent sexual transmission of mpox, PRISMA 2020 flow diagram*. *Adapted from Page et al. [25].

Using the reference management system Rayyan [11], an initial reviewer (CM) excluded duplicate records and those that did not address the research questions. Two independent reviewers (CM and IW) reviewed titles and abstracts of all remaining results. Full articles of selected abstracts were reviewed by both reviewers against the inclusion criteria. Disagreements were discussed and reconciled by consensus and with a third reviewer (SP). The PRISMA flowchart of the study selection process is shown in Figure 1.

#### Data collection process

Two reviewers (CM and IW) independently extracted study data, including study design, population (including demographic data; HIV and MPVX vaccination statuses where available), intervention details, outcome measures, key outcomes and limitations.

#### Risk of bias assessment

We assessed the risk of bias using tools developed by the CLARITY Group at McMaster University for case-control and cohort studies [12,13]. Two reviewers, CM and IW, conducted independent assessments and collaboratively resolved any conflicts.

#### Synthesis

A narrative synthesis of the available evidence is presented. Due to heterogeneity across studies, we could not perform a meta-analysis.

### Qualitative evidence synthesis

PubMed and APAPsycInfo were searched for qualitative papers reporting on primary research from 1 January 2022 to 16 January 2024. We reviewed conference abstracts from 2022 and 2023, where available online, for the following relevant meetings: International AIDS Conference; European AIDS Conference; Conference on Retroviruses and Opportunistic Infections; British HIV Association (BHIVA); British Association for Sexual Health and HIV (BASHH).

#### Search strategy

The search strategy was kept intentionally broad to allow for inclusion of all potentially relevant qualitative studies. For the full search strategies for all databases, see Appendix C.

#### Selection process

An initial reviewer (RH) excluded duplicate records and those that did not address the research question (What are the views and experiences of interventions to prevent sexual transmission of mpox among communities at risk of infection?). RH and SP independently reviewed titles and abstracts of all remaining results against the exclusion criteria (Appendix C), then reviewed full text of selected papers and reconciled differences through discussion.

#### Quality assessment

Quality was assessed by both reviewers, using 12 criteria adapted from the methodology used for the evidence review [14]. Due to the small numbers, all identified studies were included in the review.

#### Data collection and synthesis process

The degree of authors’ interpretation in the qualitative studies varied, with some adopting a descriptive narrative with few second-order constructs [15]. For our synthesis, themes identified by the study authors were extracted from the studies, and then further detail from the original texts was iteratively added to identify the key concepts within each study. Extracted data were synthesised thematically: study themes were grouped into ‘descriptive’ themes and then further synthesised to develop ‘analytic’ themes.

## Results

### Systematic review

The initial search found 1,379 results from databases and 25 conference abstracts. Three papers were identified by forward and backward citation tracking. A further 922 papers were screened and 8 were added when the search was repeated on 11 January 2024 including reports from China. Overall, a total of 17records were included in this review, including 1 conference abstract.

Manuscripts almost exclusively described higher income countries, and were heterogenous in design, with varied populations and outcomes of interest. No studies evaluated interventions to reduce onward transmission for people with confirmed mpox (question 2). Characteristics of included studies are detailed below for questions 1 and 3.

We found no randomised controlled trials, and six prospective cohort studies. The remaining papers were retrospective observational studies. For quality assessment, see Appendix D.

#### Results for question 1: do preventive interventions decrease the risk of MPVX infection?

We identified three studies meeting the inclusion criteria for question 1 (see Table 1). One retrospective observational study conducted in the US [16] examined contact tracing, while the other two cross-sectional studies [17,18] investigated specific sexual behaviours. All three studies included exclusively male participants, predominantly MSM, who self-reported their sexual behaviour. Sample sizes ranged from 472 to 7538 participants.

**Table 1.** Characteristics of included studies for question 1: do preventive interventions decrease the risk of MPVX infection?

Source	Location	Study type	Study period	Population (age range)	Sample size	Preventive intervention	Outcome measure(s)	Results
Cope et al. [16]	United States	Cross sectional	May–Jul 2022 (2 months)	Males aged ≥18 years	1921	Contact tracing	Number of reported cases, the proportion of people interviewed, the number of named contacts, named sexual contacts, unnamed contacts, and contact indices before and after vaccine campaign extension	1986 mpox cases were reported in MSM (240 before and 1746 after the expanded vaccine access). Most MSM with mpox were interviewed 228 (95%) before and 1694 (97%) after vaccination campaign. The proportion of cases who named at least one contact declined between the two periods from 75% to 40%
Ghosn et al. [17]	France	Retrospective observational	May 2022 to September 2022	Males with a median age of 39 years	472	Sexual behaviour change	Prevalence of mpox; vaccination status; self-reported behaviour change	Mpox prevalence was 16.3% (77/472) Significant change in sexual behaviour among mpox cases and mpox-free control subjects with >10 partners during the last three months(45% vs 38%, *p* = .0035)
Du et al. [18]	China	Cross sectional	July 31 to August 4, 2023 (one week)	Males (18–78 years old)	7538	Sexual behaviour change	Prevalence of mpox; crude and adjusted prevalence of infection based on a safe sexual behaviour index based on three ‘risk’ behaviours including condomless anal intercourse, commercial sex, and group sex (High ‘safe’ sexual behaviour involved participation in none, and low ‘safe’ behaviour involved engagement in all three).	Overall prevalence was 0.73% (55/7538); Crude prevalence was 0.35% (21/6003) in high ‘safe’ behaviour; 1.78% (26/1469) in moderate behaviour; and 12.12% (8/66) in low ‘safe’ behaviour. Multivariable-adjusted analysis comparing low ‘safe’ behaviour to moderate behaviour was aOR = 0.21; 95% CI = 0.08, 0.54; while comparing to high ‘safe’ behaviour was aOR = 0.04; 95% CI = 0.02, 0.12).

The study by Cope et al. [16] investigated the effectiveness of contact tracing in curbing mpox transmission among MSM, particularly after the expansion of access to mpox vaccines. They found a significant reduction in proportion of MSM with mpox who provided location details for their sexual contacts after the vaccination campaign began. This resulted in lower contact identification rates later on in the outbreak and once the vaccination campaign had taken place. The study authors suggested that increased workload on healthcare providers combined with an initial surge in reported contacts (possibly linked to desire to access vaccines) created an unsustainable baseline for later tracing efforts. This suggests that solely relying on contact tracing may be insufficient to curb mpox transmission.

The study by Ghosn et al. [17] was part of an ongoing randomised open-label trial for a sexually transmitted infection treatment in MSM, coinciding with the onset of the mpox outbreak in France when the first case was identified on 19 May 2022. Subsequently, on 11 July 2022, the mpox vaccine was recommended for all MSM and at-risk populations. The study aimed to assess mpox incidence among at-risk MSM in the trial before and after vaccination, identify mpox-associated characteristics, evaluate vaccine effectiveness, and analyse self-reported sexual behaviour modification on mpox incidence including number of sexual partners in the last 3 months. Additionally, the study compared these characteristics between mpox cases and mpox-free controls in the study. Among the 472 participants included in the study, 16.3% (77) experienced mpox, with only one case occurring after the vaccination campaign began, and the vaccination status for this case was unknown. Following the introduction of the vaccine, vaccination coverage increased from 0% to 87%, and a significant decrease in the number of sexual partners in the mpox-free control group was observed both before and after the vaccination campaign. However, adjusted analysis revealed that only the introduction of the vaccine significantly reduced mpox infections. The study authors concluded that sexual behaviour change did not appear to reduce mpox incidence in that population.

Du et al. [18] investigated the association between sexual behaviour and mpox prevalence amongst men. They developed an index of safe sex behaviour based on three behaviours: condomless anal sex, commercial sex, and group sex. Participants (*n* = 7538) were categorised into three groups: high safe sexual behaviour (no engagement in any of the behaviours), moderate safe behaviour (engagement in one or two behaviours), and low safe behaviour (engagement in all three behaviours). The overall prevalence of mpox was found to be 0.73%. Upon further analysis, the crude mpox prevalence was lower in the high safe sexual behaviour group (0.35%) compared to both the moderate (1.78%) and low (12.2%) safety sexual behaviour groups. Adjusted analysis revealed that participants in the moderate safe sexual behaviour group had a 79% lower risk of mpox compared to those in the low safe sexual behaviour group. Additionally, participants in the high safe sexual behaviour group had a 96% lower risk of mpox compared to those in the low safe sexual behaviour group. In departure from the previous study, the authors concluded that reducing engagement in low safe behaviour is important to controlling transmission.

#### Results for question 3: does testing asymptomatic individuals at increased risk of mpox without known exposure to MPXV reduce onward transmission?

A total of 13 studies including 5824 participants, (range 53 to 1,645), described asymptomatic testing for MPXV (Table 2) across Europe [19–27], USA [28,29] and Asia [30,31]; 11 studies used real-time polymerase chain reaction (PCR) testing.

**Table 2.** Characteristics and findings of included studies for question 3: does testing asymptomatic individuals at risk of mpox without known exposure to MPXV reduce onward transmission?.

Source	Location	Population	Sample size	Age range (years)	Study period	Outcome measure	Outcome/results
Adamson et al. [30]	Vietnam	MSM^a^ in HIV PrEP programme	152 participants	Median 25·9 years (IQR 21·7–30·3)	Jul–Aug 2022 (retrospectively one month)	Population mpox prevalence	None tested positive
Agusti et al. [23]	Spain	Highly exposed MSM, non-binary individual sand transgender women in community setting	113 participants	Median 35 years (IQR 30–43)	Aug–Oct 2022 (three months prospective)	Population mpox prevalence	7 participants tested positive; 8 samples tested positive. Study estimated a total prevalence of 6.19% (95% CI: 1.75–10.64%)
Contag, Renfro, et al. [29]	United States	Participants undergoing STI testing in county public hospital and clinics	1645 (participants); 1848 (swab samples)	7–81 years	Apr–Sept 2022 (five months–retrospective)	Population mpox prevalence and presence of MPXV in swab samples	11 (0.7%) participants tested positive; 16 (0.9%) samples tested positive (4/60 anorectal, 4/66 oropharyngeal, 5/1264 urine, and 3/445 vaginal)
Contag, Lu, et al. [28]	United States	Participants undergoing GC/CT testing in tertiary academic medical centre	206 participants; 347 swab samples	7 days to 77 years	Jul–Sept 2022 (two months – retrospective)	Population mpox prevalence and presence of MPXV in swab samples	17 (8%) participants tested positive; 24 (7%) samples tested positive (11/24 oropharyngeal, and 13/24 anorectal)
De Baetselier et al. [19]	Belgium	Men undergoing anorectal or oropharygneal GC/CT testing in sexual health clinic	224 participants	NR	May 2022 (retrospectively one month)	Presence of MPXV	4 (1.8%) undiagnosed cases
Ferré et al. [20]	France	Men attending routine PrEP or HIV clinic	213 participants; 200 samples analysed	Median 38 years (IQR, 29–48)	Jun–Jul 2022 (retrospectively one month)	Presence of MPXV	13 (6.5%) tested positive
Grabmeier-Pfisterhammer et al. [24]	Austria	Asymptomatic MSM at high risk for STIs attending routine screening	90 participants	Median 38 years	Jan–Mar 2023 (retrospective)	Presence of MPXV	No MPXV positive samples
Mizushima et al. [31]	Japan	Asymptomatic MSM in community setting	1346 participants	Median age 38 (IQR 31–47)	Jan–Mar 2023 (prospectively 3 months)	Presence of MPXV	5 participants (0.37%; 95% CI 0.12–0.86) positive; 3/5 remained asymptomatic
Pestel et al. [21]	Germany	MSM with changing sexual partners (PrEP-, PEP users and PLWH)	53 participants	Median 35 years (range 19-52)	Jun–Jul 2022 (prospectively one month)	Presence of MPXV	None tested positive
Pitt-Kendall et al. [25]	England	MSM undergoing routine asymptomatic GC/CT screening	1159 participants; 2927 samples	NR	Aug–Oct 2022 (retrospective)	Population MPXV prevalence	Four specimens (4/2917, 0.14%) from two individuals (2/1158, 0.17%) positive
Rossotti et al. [26]	Italy	Asymptomatic MSM PrEP users and PLWH	72 participants	Median 37 years (IQR 32–46)	Sept–Oct 2022 (prospective)	Population MPXV prevalence	None tested positive for MPXV during the initial screening or developed symptoms during the 21-day follow-up
Thomassen et al. [27]	Denmark	MSM PrEP users undergoing routine STI screening during PrEP consultations or walk-in STI testing	224 participants	Median 36.5 years (IQR 30–45)	Sept–Oct 2022 (prospective)	Presence of MPXV	1/224 participants (0.45%) positive for MPXV (developed symptoms 3 days later)
Van Dijck et al. [22]	Belgium	MSM with HIV and PrEP users	327 participants (146 retrospective; 181 prospective)	NR	Jun–Jul 22 (one month retrospectively); Jul–Sept (two months prospectively)	Population MPXV prevalence	14 (4.3%) tested positive; 3 presymptomatic; none were asymptomatic

^a^CT: *Chlamydia trachomatis*; GC: *Neisseria gonorrhoeae*; HIV: human immunodeficiency virus; IQR: interquartile range; MSM: men who have sex with men; MPXV: monkeypox virus; NR: not reported; PLWH: people living with HIV; PEP: HIV post-exposure prophylaxis; PrEP: HIV pre-exposure prophylaxis; STI: sexually transmitted infection.

Four studies assessed samples from multiple clinics across a city or region [25, 27, 29, 31]; six were in single clinical sites [19–22,24, 28], and two in community centres [23,26]. One study from Vietnam was conducted within an HIV PrEP program [30]. Seven [19,20,24,25,28–30] were retrospective, observational studies that examined stored samples taken for testing for sexually transmitted infections.

Five prospective observational studies assessed presence of MPXV in individuals at higher risk of acquiring mpox [21,23,26,27,31]. One study both examined stored samples and tested individuals attending a sexual health clinic [22].

All studies included anorectal swabs, most included oro-pharyngeal swabs [19,21–23,25,26,28,31] and one study analysed samples from multiple sites including oropharyngeal, skin lesion, urethral, vaginal, and cervical swabs [28].

The outcomes reported either the number of asymptomatic individuals with MPXV infection or the presence of MPXV in the swab samples. Only three studies [23,28,29] evaluated both male and female participants for presence of MPXV in the swab samples. Included participants ranged from 7 days to 81 years of age (one study included a neonate [29]).

Four studies [21,24,26,30] did not identify any MPXV-positive cases, and seven studies [19,20,22,23,25,27,31] reported prevalence in their respective study populations (range 0.17%–6.5%). Several studies identified presymptomatic and asymptomatic infection: Mizushima et al. [31] report that of five positive results from 1346 participants, three remained asymptomatic, one developed symptoms three days after testing, and one later reported that he had had mild symptoms prior to sample collection; Thomassen et al. [27] report one positive result of 224 participants, who developed symptoms three days later; Agusti et al. [23] report 7 positive results among 113 subjects, of whom 2 remained asymptomatic throughout follow up.

Two studies [28,29] reported MPXV prevalence in both individuals and swab samples, with prevalence of 0.7% and 8% respectively in individual samples; and MPXV prevalence of 0.9% and 7% respectively in swab samples. Oropharyngeal and anorectal swab samples showed the highest yield.

Two studies [23,27] found that asymptomatic screening was feasible and acceptable to study participants, including one [23] that found that self-sampling was feasible and acceptable to a MSM with higher exposure to mpox and transgender women in Spain.

### Qualitative evidence synthesis findings

After screening, four qualitative interview-based studies were found to have addressed the question: ‘What are the views and experiences of interventions to prevent sexual transmission of mpox among communities at risk of infection?’ Study characteristics for three cross-sectional [32–34] and one longitudinal [35] are given in Table 3. Two European studies collected data in the initial phases of the outbreak [33,34]; one study in Australia was conducted after the initial outbreak [35]; and one in China was conducted at a time when reported cases were still increasing [32].

**Table 3.** Characteristics of studies included in qualitative evidence synthesis.

Source	Location	Study aim and design	Population	Sample	Data collection period	Key findings / themes	Quality score (/12)
Zhang et al. [32]	China	To investigate the experiences of mpox patients from infection to treatment. Multicentre, cross-sectional, qualitative design. Semi-structured interviews were conducted by telephone and analysed using the thematic analysis.	Mpox patients quarantined in hospital, at home, or discharged.	15 male mpox patients. Median age = 30 (IQR: 25.5, 36), MSM (14/15), urban (15/15), bachelor’s degree or higher (9/12), monthly income ≥ CNY 5000 (10/15), HIV positive (8/15).	6–25 July 2023.	Pre-diagnosis experience: mild rashes and fever as common symptoms; limited knowledge of mpox; sex as most likely route of infection Treatment-seeking experience: delays seeking treatment due to mildness of symptoms; difficulties getting accurately diagnosed; attitude towards diagnosis Quarantine experience: damaging effects of hospital quarantine; reluctance to self-disclose infection status due to stigma and potential discrimination; importance of family support for recovery. Advice for mpox prevention and control: increase in testing channels and methods; development and introduction of vaccines; adjustments to quarantine program; improvement of treatment measures; improvement of publicity and education	7/12 (medium)
Vanhamel et al. [33]	Belgium	To describe the initial phase of the outbreak in Belgium and to provide a more in-depth description of sexual behaviour and transmission contexts. Mixed methods study, combining national routine surveillance data and narrative data from individual, semi-structured interviews. Interviews were held *via* telephone or online.	Individuals with laboratory-confirmed mpox cases with onset of symptoms between 10 May and 19 June 2022.	12 interview participants, all men, and all aged over 30.	24 May − 20 June 2022.	Self-perceived exposure settings and contexts: gay-oriented festival; cruising venue; home; unknown (sexual); unknown (non-sexual). Sexual interactions: anonymous encounters reduce opportunities for contact tracing; mpox-suspected symptoms among sex partners; sexual networks of MSM. Health-seeking behaviour and risk perception related to mpox: confusing mpox symptoms for other STIs; confusing mpox symptoms for other skin conditions; risk perception; provider-related diagnostic delay.	6/12 (medium)
May et al. [34]	UK	To describe knowledge, attitudes and perceptions of a sample of MSM towards mpox during the 2022–2023 outbreak in the UK, including facilitators for and barriers to the uptake of transmission-reducing behaviours; and to explore preferences for optimising and adapting public health messaging to improve the uptake of measures. Qualitative, cross-sectional design using semi-structured remote telephone/video-call methods.	People with confirmed mpox in 2022, and MSM. All aged >18 and living in UK.	Interviews were conducted with 44 MSM, average age = 34.2 (range 20–56). White British (*n* = 21), White Other (*n* = 6), Black / African / Caribbean (*n* = 5), Latin American (*n* = 5), Asian (*n* = 3), Other ethnic group (*n* = 2), White Welsh (*n* = 2). 13/44 reported recent contact with a confirmed mpox case, and 8/44 reported recent mpox diagnosis.	May–December 2022.	Perceived risk and severity of mpox influenced willingness to engage with some measures, e.g. healthcare seeking and vaccination uptake. Perceived acceptability of and capacity to perform measures: sexual health-related knowledge, practices, and support; stigma and sexual orientation openness; retaining intimacy; (they also include a table outlining barriers to specific measures, which include structural constraints such as vaccine availability and financial implications of isolation). Optimisation of messaging: targeted vs. inclusive messaging; focus on severe symptoms in messaging could mean that early infection goes unnoticed; Lack of information regarding mpox symptoms, including potential symptom overlaps and information on how to differentiate from other STIs; Lack of targeted messaging for those at increased risk; Absence of harm reduction messages; Official posters/ messages not as credible as those from within MSM community; Absence of information around looking after yourself (self-care) with mpox.	10.5/12 (high)
Smith et al. [35]	Australia	To explore the social and health experiences of people affected by mpox and understand how people with mpox can be better supported in healthcare. Longitudinal, qualitative design using two semi-structured interviews, 6 months apart.	People diagnosed with mpox in 2022, or close contacts.	16 participants, 13/16 diagnosed with mpox. 16/16 cisgender MSM living in Australia.	First interviews: Oct–Dec 2022. Follow-up interviews: Apr–May 2023.	Narrating acute illness and long-term effects: minor to severe periods of sickness, negative and stigmatising experiences engaging with healthcare (with good care as the exception), some participants experienced long-term effects on their sexual wellbeing and complications from mpox. The emergency context in which mpox emerged could make the disease highly distressing and difficult to manage, and produced varying forms of disruption to everyday life. Mpox was narrated as disruptive in different ways: as a minor interruption to holiday plans; a prolonged period of poor health; or as an event prompting a re-evaluation of sexual values and health.	11/12 (high)

Three studies [32,33,35] only included men (mostly MSM) with experience of mpox. The fourth study [34] included only MSM.

All studies included participants’ views on mpox prevention measures, focusing particularly on: behaviour prior to the onset of symptoms [33], behaviour and practices from the time of symptom onset to recovery [32], participants’ experiences of treatment and care [35], and engagement in primary and/or secondary prevention measures [34].

Evidence synthesis resulted in four the mes: *provision of accurate information on mpox*; *the diversity of sexual practices and settings*; *the importance of accessible and high-quality mpox testing and patient care for secondary prevention*; and *how acceptability of an intervention relates to the perceived cost to wellbeing*.

#### Effective mpox prevention depends on the provision of accurate information

Participants understood sex to be the primary transmission route for mpox, and that increased sexual activity and in particular settings could increase risk of mpox acquisition [32–34]. However, one study [33] highlighted that network-level factors that made MSM especially susceptible to mpox were unclear to participants. This meant that those who limited their sexual activity perceived themselves as having low risk of infection (and, in the case of those who acquired mpox, did not immediately recognise their symptoms).

All four studies highlighted that a lack of information about the range of mpox symptoms made self-diagnosis challenging for participants who acquired mpox: mpox symptoms, especially when mild, were confused with other STIs or skin conditions, leading to delays in seeking treatment. Perceptions of mpox severity and ability to treat mpox also influenced people’s experiences of mpox and their decisions to engage in prevention behaviours.

#### Not all sexual practices and settings afford opportunities for standard prevention approaches

Participants discussed how having sex with anonymous or unknown partners meant acquiring details for contact tracing which could be difficult or impossible, and the expectations of ‘contact tracers’ were unrealistic [33,34]. Similarly, checking sexual partners for possible mpox symptoms was also seen as impractical. Participants considered abstinence-based measures unfeasible, preferring safer sex and risk mitigation advice [34].

#### MSM are motivated to engage in prevention measures, but accessibility and quality of testing and patient care may reduce engagement

All study authors indicated that MSM taking part in their studies were highly engaged with sexual health services and motivated to seek testing and care for possible STIs. However, there were challenges in accessing testing and accurate diagnosis, necessitating multiple visits and insistence on specialist referrals at times.

Once diagnosed, many participants felt that the predominant focus of clinicians was infection control, rather than care [32,35]. Participants in China found mandatory hospital quarantine excessive in the context of lack of mpox treatment, and that self-isolation at home would have been preferable [32]. Self-isolation was seen as particularly challenging for those in precarious employment, and for those in shared accommodation [32,34].

#### Acceptability is relative to perceived cost for individuals’ wellbeing

Three studies [32,34,35] described acceptability of different mpox prevention measures, indicating that acceptability of a measure was relative to the participant’s perception of its cost to their wellbeing.

Participants weighed up the risks of acquiring mpox against cost to their wellbeing from engaging in prevention measures, which was influenced by their experience of other health issues, the perceived severity of mpox(including concerns about scarring), and their degree of knowledge about mpox [32,34,35]. Those considering sex-on-premises spaces as central to their social and sexual lives deemed it unfeasible to avoid sex altogether [34].

Concerns about mpox stigma were a barrier to engagement in prevention measures requiring disclosure (e.g. testing, contact-tracing) [32,34]. Anticipated mpox stigma was related to individuals’ feelings about their sexuality–some were worried about engaging in secondary prevention measures which could lead to direct or indirect disclosure of their sexuality or to them being labelled as ‘promiscuous’. In May et al. some participants described their prevention measures to avoid disclosure – for example, attending work while symptomatic but once any visible symptoms (i.e. on face, arms, etc.) had passed [34].

Lastly, participants with experience of mpox noted that emotional and practical support from family and friends, and positive experiences of care from clinicians, were vital to recovery and to engaging in prevention measures such as self-isolation.

## Discussion

Behaviour change adaptations and other public health interventions were recommended to reduce transmission of mpox [10], including prior to and following mpox vaccine introduction in some settings. The search strategy for this review was thus formulated to assess the benefits of a range of preventive interventions other than vaccines and perceptions of them among persons at risk. Research beyond this review does suggest that those at risk of acquiring mpox have been modifying their behaviour in response to the outbreak [32–38]. For some, these changes have endured: a WHO survey of people at increased risk of mpox acquisition found 35% of participants who had changed their sexual behaviour because of mpox concerns said they were still doing so a year after the outbreak was identified [39].

However, our review has so far found limited quantitative evidence evaluating the *effectiveness* of preventive interventions on sexual transmission of mpox, for either acquisition or onward transmission. This may reflect the challenges in conducting evaluation of behaviour change and other public health interventions during a rapidly developing outbreak of an emerging – and in some contexts, poorly understood - infectious disease.

We found only limited data on the benefits of contact tracing [16], with the sharing of information about contacts by patients declining once vaccines become available. Furthermore, qualitative evidence offers important context regarding the limits of contact tracing due to concern about stigma and in some instances due to the anonymous nature of some sexual contacts [33].

Evidence on the utility of asymptomatic testing(question 3) was also limited. Studies reported on the identification of MPXVDNA in persons who were asymptomatic or presymptomatic and the potential for early transmission, underscoring that there may be benefits in raising awareness and intensifying case finding during an outbreak and supporting partner notification where possible. However, there were no studies evaluating the impact of asymptomatic testing interventions in relation to overall transmission. Asymptomatic testing studies were significantly confounded by the timing in relationship to the respective outbreak, lack of controls, small sample sizes, retrospective study design, short study or recruitment periods, and recruitment from a single site. Where asymptomatic testing was seen to be feasible and/or acceptable [23,27], studies did not evaluate cost effectiveness for their respective target populations. In addition, no study identified levels of disease prevalence in which asymptomatic testing may be a useful tool.

Nonetheless, against the background of limited evaluative, quantitative studies, our qualitative evidence synthesis indicates that most affected communities were engaged with preventive and harm reduction strategies during the outbreak. Interventions that increase knowledge of mpox and reduce stigma are most helpful to prevention efforts, as are positive experiences in healthcare. Focussing interventions towards MSM through respectful and non-discriminatory partnering with MSM community organisations and promoting peer-to-peer information sharing was considered reasonable by participants.

### Limitations and conclusions

This review is subject to several limitations. The evidence was largely descriptive, rather than evaluative. The heterogeneity of study design and findings made pooling data unfeasible. Studies answering Question 3 relied mostly on self-reporting of symptoms, and there were possible under-estimates of asymptomatic transmission where studies were conducted after the peak of the outbreak in their country. This led to later prospective studies included and finding of low prevalence of asymptomatic infection. Finally, while there were only four qualitative studies addressing our question, these were from significantly different geographic contexts, enhancing global understanding of themes identified.

The primary focus of this systematic review was finding evidence about strategies to reduce sexual transmission of mpox. As a result, the published literature predominantly emerged from non-endemic regions experiencing new outbreaks related to sexual transmission and most of the reviewed literature came from higher income settings. This may limit the transferability of our findings to lower-resource settings and countries where mpox is endemic. As the epidemiology of mpox globally changes however, we believe our findings can have relevance in any context where mpox is transmitted through sexual contact, particularly amongst MSM.

In conclusion, although the mpox public health emergency of international concern was declared over in May 2023, cases continue to be reported worldwide, with the highest number of confirmed cases in the WHO Region of the Americas and the European Region [40]. Many lower and middle-income countries including endemic countries in Africa still do not have sufficient diagnostic capacity or any access to vaccines or antivirals outside clinical trials. There is a continuing need for global co-operation to ensure equity of access to diagnostics, vaccines and antivirals to all who need them.

Neither natural immunity from previous MPXV infection nor post-vaccination immunity are entirely effective at protecting individuals from MPXV re-infection, although they may limit illness duration and severity [41]. WHO has highlighted the need for further research to better understand the underlying mechanisms in recurrent mpox disease [42].

Collectively, these issues underscore the importance of identifying other effective preventive measures for preventing transmission in different contexts which are also acceptable to communities at risk of mpox. Working with communities to improve mpox education is important to support decision-making around risk and finding equitable ways to reduce the perceived and actual social and economic costs of prevention interventions remains paramount. Such complementary measures should also be explored, such as offering harm reduction advice, providing financial or other forms of support to patients, and proposing public health measures appropriate to different local contexts.

## Note

1 ID Week is the joint annual meeting of the Infectious Diseases Society of America (IDSA), Society for Healthcare Epidemiology of America (SHEA), the HIV Medicine Association (HIVMA), the Pediatric Infectious Diseases Society (PIDS), and the Society of Infectious Diseases Pharmacists (SIDP).

## Appendix A – PICO questions

**Table A1.** Question: do preventive interventions decrease the risk of mpox infection?

	Definition
Population	Persons at increased risk^a^ of acquiring mpox through sexual contact (e.g. sexual networks or casual, multiple partners)
Intervention:	Harm reduction or preventive interventions Reduction of number of sexual partners Condom use Change in types of sexual contact Evaluation of partner (e.g. asking about symptoms, inspecting for lesions)
Comparator	No interventions
Outcome	1) Acquisition of mpox infection 2) Complicated mpox infection (proctitis, urethritis and/or tonsillitis)
Subpopulations	People living with HIV; those with prior smallpox vaccination;

^a^ Individuals at increased risk of mpox acquisition through sexual contact included sexually active gay, bisexual and other men who have sex with men and people in their sexual networks, sex workers, and transgender and gender-diverse people. In the absence of epidemiological information on risk factors for mpox, this risk of exposure was proxied by a recent history of a sexually transmitted infection (STI), HIV pre-exposure prophylaxis (PrEP) use, or living with HIV.

**Table A2.** Question: does testing asymptomatic individuals at increased risk of mpox (without known exposure) reduce onward transmission?

	Definition
Population	Persons with mpox at risk of onward transmission through sexual contact
Intervention:	Harm reduction or preventive interventions: Abstinence for a given period of time Reduction of number of sexual partners Condom use Change in types of sexual contact/activity Disclosure to partners Covering of lesions
Comparator	No interventions
Outcome	Transmission of mpox infection to partner(s)
Subpopulations	People living with HIV

**Table A3.** Question: does testing asymptomatic individuals at increased risk of mpox (without known MPVX exposure) reduce onward transmission?

	Definition: Testing for asymptomatic MPXV infection among people at increased risk without known exposure
Population	Asymptomatic individuals at increased risk of acquiring and transmitting mpox through sexual contact or with history of contact with mpox patient, without known exposure
Intervention:	Testing of asymptomatic individuals at increased risk without known exposure
Comparator	Not testing asymptomatic individuals at increased risk without known exposure (testing only symptomatic individuals and asymptomatic individuals with known exposure)
Outcome	Case detection rate - increased detection of mpox infections Decreased transmission

## Appendix B – quantitative review search strategies

**Ovid MEDLINE(R) ALL <1946 to May 09, 2024>**

1‘sex*’.ab,ti. 895307

2 exp Sex/7738

3 exp Sexual Behaviour/ 123975

4 Coitus/ 7689

5 1 or 2 or 3 or 4 936624

6 (monkeypox or Mpox or ‘**monkey** pox’).ab,ti. 3206

7 exp Monkeypox virus/or exp **Monkeypox**/ 2042

8 6 or 7 3281

9 5 and 8 413

10 limit 9 to yr=‘2022 -Current’ 407

**Embase <1974 to 2023 May 09>**

1‘sex*’.ab,ti. 1235076

2 exp sex/ 437348

3 exp sexual intercourse/ 34432

4 exp sexual behaviour/ 245807

5 1 or 2 or 3 or 4 1411061

6 (monkeypox or Mpox or ‘monkey pox’).ab,ti. 3522

7 exp monkeypox/or exp Monkeypox virus/ 3466

8 6 or 7 3918

9 5 and 8 640

10 limit 9 to yr=‘2022 -Current’ 630

**PubMed**

((‘Sex’[Title/Abstract] OR (‘Sex’[MeSH Terms] OR ‘Sexual Behaviour’[MeSH Terms] OR ‘Coitus’[MeSH Terms])) AND (‘Monkeypox’[Title/Abstract] OR ‘Mpox’[Title/Abstract] OR ‘monkey pox’[Title/Abstract] OR ‘Monkeypox’[MeSH Terms] OR ‘Monkeypox virus’[MeSH Terms])) AND (2022:2024[pdat])

**Cochrane Library**

#1    (monkeypox or Mpox or ‘monkey pox’):ab,ti,kw [21]

#2    MeSH descriptor: [Monkeypox] explode all trees [12]

#3    MeSH descriptor: [Monkeypox virus] explode all trees [3]

#4    #1 or #2 or #3 with Cochrane Library publication date Between Jan 2022 and May 2023 (15)

**WHO International Clinical Trials Registry Platform (ICTRP)**https://trialsearch.who.int/mpox or monkey pox or monkeypox [35]

**Updated search results (January 2024) (Embase, Medline, Cochrane)**

1 ‘sex*’.ab,ti. 934131

2 exp Sex/7748

3 exp Sexual Behaviour/127058

4 exp Coitus/7946

5 1 or 2 or 3 or 4 975832

6 (prevent* or ‘harm reduc*’ or interven* or vaccin*).ab,ti. 3319616

7 exp Primary Prevention/185716

8 exp Harm Reduction/4230

9 exp Vaccines/283284

10 6 or 7 or 8 or 9 3422117

11 (monkeypox or Mpox or ‘monkey pox’).ab,ti. 4304

12 exp Monkeypox virus/or exp Monkeypox/1263

13 11 or 12 4357

14 5 or 10 4232111

15 13 and 14 2128

16 limit 15 to yr=‘2023 -Current’ 1096

## Appendix C – qualitative review search strategies and results

**PubMed**

((‘mpox monkeypox’[MeSH Terms] OR (‘mpox’[All Fields] AND ‘monkeypox’[All Fields]) OR ‘mpox monkeypox’[All Fields] OR ‘monkeypox’[All Fields] OR (‘mpox monkeypox’[MeSH Terms] OR (‘mpox’[All Fields] AND ‘monkeypox’[All Fields]) OR ‘mpox monkeypox’[All Fields] OR ‘mpox’[All Fields])) AND (‘qualitative’[All Fields] OR ‘qualitatively’[All Fields] OR ‘qualitatives’[All Fields] OR (‘interview’[Publication Type] OR ‘interviews as topic’[MeSH Terms] OR ‘interview’[All Fields]))) AND ((english[Filter]) AND (2022:2024[pdat]))

Extracted results: 35

**PsycInfo**

S1 monkeypox 35

S2 mpox 18

S3 qualitative242,967

S4 interview450,333

S5 S1 OR S2 41

S6 S3 OR S4 573,630

S7 S5 AND S6 1

**Conferences**

BASHH: 10

BHIVA: 4

CROI: 2

EACS: 17

IAS:21

**Screening**

Studies were excluded if they:

a) Didn’t collect or analyse primary qualitative research data.

b) Didn’t report on the views or experiences of people at risk of sexual transmission of mpox with regards to changing behaviour to prevent mpox transmission.

c) Focused exclusively on mpox vaccination.

Records screened: 90.

Records excluded: 86.

## Appendix D – risk of bias assessment

### Assessment statement and limitations

Most observational studies, evaluated using the cohort study tool, had a high risk of bias primarily because there was no comparator intervention or group nor any follow up. The tool we’ve been employing is primarily designed for cohort studies, where there are distinct exposed and non-exposed groups that can be followed either retrospectively or prospectively. However, many of the studies reviewed are either cross-sectional or single-cohort prospective or retrospective studies. Consequently, when categorising responses to the tool’s questions, there were many ‘definitely no’ responses or difficulty in classification. This is a limitation when assessing our findings, as the tool is primarily tailored for cohort studies involving two distinct groups (exposed and non-exposed).

**Table D1.** Cohort studies for question 1.

Author, year	1. Was selection of exposed and non-exposed cohorts drawn from the same population?	2. Can we be confident in the assessment of exposure?	3. Can we be confident that the outcome of interest was not present at start of study	4. Did the study match exposed and unexposed for all variables that are associated with the outcome of interest or did the statistical analysis adjust for these prognostic variables?	5. Can we be confident in the assessment of the presence or absence of prognostic factors?	6. Can we be confident in the assessment of outcome?	7. Was the follow up of cohorts adequate?	8. Were co-Interventions similar between groups?
Cope et al., 2023 [16]	Definitely no	Probably yes	Definitely yes	Definitely no	Definitely no	Probably no	Probably yes	Definitely no
Du et al., [18]	Definitely no	Probably yes	Probably yes	Mostly yes	Probably yes	Probably yes	Probably yes	Definitely no
Ghosn et al., 2023 [17]	Definitely no	Definitely yes	Probably yes	Mostly no	Probably no	Definitely yes	Probably yes	Definitely no

*Note:* Response options: definitely yes (low risk of bias); probably yes; probably no; definitely no (high risk of bias).

**Table D2.** Cohort studies for question 3.

Author, year	1. Was selection of exposed and non-exposed cohorts drawn from the same population?	2. Can we be confident in the assessment of exposure?	3. Can we be confident that the outcome of interest was not present at start of study	4. Did the study match exposed and unexposed for all variables that are associated with the outcome of interest or did the statistical analysis adjust for these prognostic variables?	5. Can we be confident in the assessment of the presence or absence of prognostic factors?	6. Can we be confident in the assessment of outcome?	7. Was the follow up of cohorts adequate?	8. Were co-Interventions similar between groups?
Adamson et al., 2023 [30]	Definitely no	Definitely yes	Definitely yes	Definitely no	Definitely no	Definitely yes	Definitely yes	Definitely no
Agusti et al., 2023 [23]	Definitely no	Probably yes	Probably yes	Definitely no	Definitely no	Probably yes	Definitely no	Definitely no
Contag, Lu, et al., 2022 [28]	Definitely no	Probably yes	Definitely yes	Definitely no	Definitely no	Probably yes	Probably yes	Definitely no
Contag, Renfro, et al., [29]	Definitely yes	Definitely yes	Probably yes	Definitely no	Definitely no	Probably yes	Probably no	Definitely no
De Baetselier et al., 2022 [19]	Definitely no	Definitely yes	Probably yes	Definitely no	Definitely no	Definitely yes	Definitely yes	Definitely no
Ferré et al., 2022 [20]	Definitely no	Definitely yes	Probably yes	Definitely no	Definitely no	Probably yes	Definitely no	Definitely no
Grabmeier-Pfisterhammer et al., 2024 [24]	Definitely no	Definitely yes	Definitely yes	Definitely no	Definitely no	Probably yes	Definitely no	Definitely no
Mizushima et al., 2023 [31]	Definitely no	Probably yes	Probably yes	Definitely no	Definitely no	Definitely yes	Probably yes	Definitely no
Pestel et al., 2022 [21]	Definitely no	Definitely yes	Definitely yes	Definitely no	Definitely no	Definitely yes	Definitely no	Definitely no
Pitt-Kendall et al., 2023 [25]	Definitely no	Definitely yes	Probably yes	Definitely no	Definitely no	Definitely yes	Definitely no	Definitely no
Rossotti et al., 2023 [26]	Definitely no	Definitely yes	Definitely yes	Definitely no	Definitely no	Definitely yes	Probably yes	Definitely no
Thomassen et al., 2024 [27]	Definitely no	Definitely yes	Definitely yes	Definitely no	Definitely no	Probably yes	Probably yes	Definitely no
Van Dijck et al., 2023 [22]	Definitely no	Definitely yes	Definitely yes	Definitely no	Definitely no	Definitely yes	Definitely yes	Definitely no

*Note:* Response options: definitely yes (low risk of bias); probably yes; probably no; definitely no (high risk of bias).
